# Host Iron Nutritional Immunity Induced by a Live *Yersinia pestis* Vaccine Strain Is Associated with Immediate Protection against Plague

**DOI:** 10.3389/fcimb.2017.00277

**Published:** 2017-06-21

**Authors:** Ayelet Zauberman, Yaron Vagima, Avital Tidhar, Moshe Aftalion, David Gur, Shahar Rotem, Theodor Chitlaru, Yinon Levy, Emanuelle Mamroud

**Affiliations:** Department of Biochemistry and Molecular Genetics, Israel Institute for Biological ResearchNess-Ziona, Israel

**Keywords:** *Yersinia pestis*, plague, iron nutritional immunity, hemopexin, live vaccine, EV76, protective immunity, innate immunity

## Abstract

Prompt and effective elicitation of protective immunity is highly relevant for cases of rapidly deteriorating fatal diseases, such as plague, which is caused by *Yersinia pestis*. Here, we assessed the potential of a live vaccine to induce rapid protection against this infection. We demonstrated that the *Y. pestis* EV76 live vaccine protected mice against an immediate lethal challenge, limiting the multiplication of the virulent pathogen and its dissemination into circulation. *Ex vivo* analysis of *Y. pestis* growth in serum derived from EV76-immunized mice revealed that an antibacterial activity was produced rapidly. This activity was mediated by the host heme- and iron-binding proteins hemopexin and transferrin, and it occurred in strong correlation with the kinetics of hemopexin induction *in vivo*. We suggest a new concept in which a live vaccine is capable of rapidly inducing iron nutritional immunity, thus limiting the propagation of pathogens. This concept could be exploited to design novel therapeutic interventions.

## Introduction

Live vaccines are able to elicit extensive humoral immunity against specific antigens as well as inflammatory and cellular protective immune responses. Furthermore, the presentation of antigens to the host immune system in the context of infection can potentiate the amplitude and longevity of the protective response.

While the ability of live vaccines to serve as platforms for the initiation of adaptive protective responses against infections has been well documented (Coward et al., 2014; Minor, 2015), their ability to evoke protective innate immune mechanisms remains largely unknown. The induction of rapid, non-specific protective responses by live vaccines could provide a means to delay the progression of infection during sudden outbreaks, thereby extending the therapeutic window and allowing for the elicitation of the adaptive response, the manifestation of which typically requires more time post-exposure. In addition, the identification of potent innate immune mechanisms could lead to the discovery of novel bacterial or host-borne therapy targets, which are essential countermeasures to antibiotic-resistant pathogens.

Plague, which is caused by *Yersinia pestis*, is a fatal disease that has caused millions of deaths in three world pandemics. Over the last few decades, plague has persisted in Africa, Asia and the Americas, and since the 1990s, it has been categorized as a re-emerging disease (Bertherat, 2016). At the same time, *Y. pestis* has been recognized as a Tier 1 select biological agent due to its potential use as a bioweapon (Inglesby et al., 2000). The severity of the disease in combination with concerns regarding the existence of antibiotic-resistant *Y. pestis* strains has prompted efforts to develop safe live vaccines (Titball and Williamson, 2004; Feodorova et al., 2007; Tidhar et al., 2009; Feodorova and Motin, 2012; Dentovskaya et al., 2015; Derbise et al., 2015; Tiner et al., 2016; Verma and Tuteja, 2016).

Protection against plague can be achieved by reiterated administrations of live EV76-based vaccines, which elicit a strong adaptive protective immune response. As of today, the only well-established molecular basis for the attenuation of these vaccines is attributed to the absence of the pigmentation locus (*pgm*)-which results in iron acquisition deficiency, a process essential for the survival and expansion of the pathogen in the host (Fetherston et al., 1992; Perry and Fetherston, 1997). Whether live vaccines such as EV76 are merely attenuated mutants or whether they manifest protection-inducing activities that are silent in their virulent parental strains remains an unanswered issue.

In the current report, we evaluated the efficacy of the *Y. pestis* EV76 live vaccine strain in providing immediate protection against both bubonic and pneumonic plague, and we characterized the mechanism involved in this innate immune response.

## Materials and methods

### Bacterial strains

The *Y. pestis* strains used in this study included Kimberley53 (Kim53), a fully virulent *Y. pestis* strain (biovar orientalis), the live vaccine strain EV76 (*pgm*^−^ [Girard's strain]) and the avirulent Kim53Δ70Δ10 strain (Ben-Gurion and Shafferman, 1981; Flashner et al., 2004; Zauberman et al., 2013). Kim53Δ70Δ10 strain was spontaneously cured of pCD1 (carrying the genes coding for elements of the Type III secretion system, essential for manifestation of virulence, Perry and Fetherston, 1997) and pPCP1 (carrying the gene coding for the virulence factor plasminogen-activating protease, Sebbane et al., 2006; Lathem et al., 2007).

To construct a bioluminescent *Y. pestis*, the plasmid pGEN-luxCDABE (a generous gift from Professor H. Mobley, Lane et al., 2007) was introduced into the *Y. pestis* Kim53 strain by electroporation, generating the Kim53-lux strain. Maintenance of the virulence-associated plasmids pMT1, pCD1, and pPCP1 and the *pgm* locus in the Kim53-lux strain was verified by PCR analysis.

### Ethics statement

This study was performed in strict accordance with the recommendations in the Guide for the Care and Use of Laboratory Animals from the National Institutes of Health. All of the animal experiments were performed in accordance with Israeli law and were approved by the Ethics Committee for animal experiments at the Israel Institute for Biological Research. (Protocols M-03-14, M-28-14, M-47-15, M-20-16, and M-37-16).

### Animal studies

Female 8 to 12-week-old C57BL/6 mice (Harlan, Israel) were used in this study. For s.c. infections in the mice, *Y. pestis* bacteria were grown on brain heart infusion agar (BHIA, Difco) for 48 h at 28°C and were then suspended in saline solution (0.9% NaCl). Protection experiments were carried out by challenging vaccinated animals by two routes of infection-s.c. or i.n. (i) Mice were infected simultaneously with 1 × 10^7^ CFU of EV76 in the upper back (s.c. vaccination) and with 100 CFU (100 LD_50_), of Kim53 in the lower back (s.c. challenge). (ii) Mice were infected with 1 × 10^7^ CFU of EV76 in the upper back (s.c. vaccination) simultaneously or 2 days prior to i.n. infection (challenge) with 1 × 10^4^ CFU (10 LD_50_) of Kim53. Mice infections were performed as described previously (Zauberman et al., 2009; Vagima et al., 2015). Bacteria were quantified by CFU counting on BHIA plates. For bacterial dissemination to internal organs and blood, groups of at least 4 mice were anesthetized; tail vein blood was collected; spleens, draining inguinal lymph nodes (I-LNs), draining axillary lymph nodes (Ax-LNs) and mediastinal lymph nodes were harvested; and then tissue homogenates were prepared in 1 ml of PBS/organ. Bacterial quantification in the tissue homogenates or in blood samples was performed by plating serial dilutions in PBS on BHIA and by calculating the CFU/organ or CFU/1 ml of blood. To differentiate between EV76 and Kim53 in the organs, the samples were plated on BHIA supplemented with 100 μg/ml streptomycin, conditions that allow for the growth of only the Kim53 streptomycin-resistant strain.

### Whole-animal luminescent imaging

Anesthetized mice previously infected s.c. with 100 CFU of Kim53-lux were analyzed by visualizing photon emission using an *in vivo* Imaging System-IVIS (Caliper Life Sciences, Hopkinton, MA, USA). Image acquisition was performed with the binning set to 2. The acquisition time ranged between 1 and 4 min. The luminescence signals for all of the images were normalized using Living image® 4 software (PerkinElmer, USA) and are reported as photons/second/cm^2^/sr.

### *Ex vivo Y. pestis* growth assay

For *ex vivo* growth assays, mouse serum were mixed with 1 × 10^3^ *Y. pestis* bacteria (total volume of 100 μl) and incubated in microtiter plates at 28°C. Dilution of mouse serum was done in RPMI medium supplemented with 10% fetal calf serum. Test samples and control samples contained identical amounts of mouse serum. Kim53 or Kim53Δ70Δ10 bacterial growth was determined by serial dilution and viable cell counts. The inhibitory effect was similar in both strains. To assess bacterial growth in immunized mouse serum in the presence of iron, iron dextran (Sigma D8517) was added at concentrations between 1.3 μg/ml to 1 mg/ml in 3-fold serial dilutions.

### Proteinase K digestion

Proteolysis was performed by incubating the serum samples with proteinase K-conjugated beads (Sigma Aldrich P9290) for 2 h at 37°C according to the manufacturer's recommendations. Proteinase K-treated serum samples were centrifuged to remove the beads, and the supernatants were collected for analysis.

### SDS-Page, western blot analysis and Anti-F1 Elisa

Serum proteins (10 μl) were separated by 4–12% gradient SDS-PAGE (Invitrogen) and were stained using a Silver Stain Plus kit (Bio-Rad). For Western blot analysis, electrophoresed samples were transferred to nitrocellulose membranes (Invitrogen). Membranes were developed with polyclonal antibodies against hemopexin (Abcam ab 90947), transferrin (Abcam ab 82411) and β-actin (Sigma A5060), followed by HRP-conjugated goat anti-rabbit IgG. Anti-F1 ELISA in the serum of immunized mice was performed as described previously (Levy et al., 2011, 2016). Briefly, microtiter plates were coated with 500 ng of purified rF1 [provided by the Biotechnology Department at IIBR, produced as described in Holtzman et al. (2006)]. Tested sera were serially diluted in 2-fold dilutions in a final volume of 50 μl and were incubated in the wells for 1 h at 37°C. Alkaline phosphatase-conjugated goat anti-rabbit IgG (1/2,000 dilution, Sigma) was used as the 2nd layer for rabbit anti-F1 IgG titer determination. Titers were defined as the reciprocal values of the endpoint serum dilutions that displayed OD_405_ values 2-fold higher than the normal serum controls.

### Ion-exchange chromatography

A pooled serum sample from EV76-infected mice (48 h post-infection) was first diluted and filtered through a 100 k-Da size exclusion membrane (VIVASPIN, Sartorius). The *Y. pestis ex vivo* growth inhibitory activity was identified to be in the upper fraction. The upper fraction was diluted 1:5 in buffer A (25 mM HEPES, pH 8.0) and was loaded onto a HiTrapQ HP 5-ml column from GE Healthcare equilibrated in the same buffer, using an AKTA Explorer system (GE-Healthcare) at 4°C. After loading, the column was washed with 10 column volumes (cv) of 100% buffer A at 4 ml/min, and it was eluted using a composite gradient between buffer A and B (25 mM HEPES, pH 8.0, and 1 M NaCl) with 20 cv of 10–30% B, 2 cv of 30% B, and 3 cv of 30–100% B at 2 ml/min, collecting 2 ml fractions. Finally, the column was eluted with 3 cv of 100% B at 2 ml/min, collecting 4 ml fractions. The fractions were sampled and used for *ex vivo* bacterial growth assays.

The fraction containing the inhibitory growth activity was subjected to a second purification step using anion exchange chromatography. After loading the sample, the column was washed with 5 cv of 100% A at 2 ml/min, collecting 4 ml fractions, and was eluted using a composite gradient between buffer A and C (25 mM HEPES, pH 8.0, and 150 mM NaCl) with 20 cv of 0–15% C at 1 ml/min, collecting 2 ml fractions. Then, the elution was continued with 5 cv of 15% C, 4 cv of 20% C, 3 cv of 25% C, 3 cv of 25–100% C and 3 cv of 100% C at 2 ml/min, collecting 2 ml fractions. Finally, the column was eluted with 3 cv of 100% C at 2 ml/min, collecting 4 ml fractions. The fractions were sampled and used for *ex vivo* bacterial growth assays. Faction 26 from the second chromatographic purification step exhibited the highest growth inhibitory activity.

### Mass spectrometry

Fraction 26, which contained most of the growth inhibitory activity, was subjected to in-solution digestion and was partially dried in an Eppendorf SpeedVac, and the remaining sample was resuspended in 100 mM ammonium bicarbonate to a final volume of 50 μl. Prior to enzymatic digestion with trypsin, the sample was reduced with dithiothreitol (DTT) and alkylated with iodoacetamide. Briefly, reduction was conducted at a final concentration of 10 mM DTT at 56° for 1 h. Iodoacetamide was then added to a final concentration of 55 mM, and the reaction was incubated at room temperature for 30 min. Finally, the reaction was quenched with 10 mM DTT. Enzymatic digestion was conducted overnight at 37°C with trypsin at an enzyme to protein ratio of 1:50 (wt/wt). The sample was partially dried in a SpeedVac. The resulting peptide mixtures were diluted in 80% formic acid and were then diluted 1:10 with Milli-Q water immediately before inline reverse-phase nano-LC (liquid chromatography)-electro spray ionization (ESI) tandem mass spectrometric analysis (MS/MS).

Samples were analyzed using an LTQ Orbitrap (Thermo Fisher Scientific, Bremen, Germany) operated in the positive ion mode. Nano-LC-ESI-MS/MS peptide mixtures were separated by inline reversed-phase nanoscale capillary LC and were analyzed by ESI-MS/MS. For LC-MS/MS, the samples were injected onto a 15 cm reversed-phase spraying fused-silica capillary column [constructed in-house; inner diameter 75 μm and packed with 3 μm of ReproSil-Pur C18A18 media (Dr. Maisch GmbH, Ammerbuch-Entringen, Germany)] using an UltiMate 3000 Capillary/Nano LC System (Dionex). The LC setup was connected to an LTQ Orbitrap mass spectrometer (Thermo Fisher Scientific, Bremen, Germany) equipped with a nanoelectrospray ion source (Thermo Fisher Scientific, Bremen, Germany). The flow rate through the column was 300 nl/min. An acetonitrile gradient was employed with a mobile phase containing 0.2% formic acid in Milli-Q water. The injection volume was 5 μl. The peptides were separated by 4 h gradients that ranged from 5 to 65% acetonitrile. The voltage applied to the union to produce an electrospray was 1.2 kV. Helium was introduced as a collision gas at a pressure of 3 psi. The mass spectrometer was operated in the data-dependent mode. Survey MS scans were acquired in the Orbitrap with the resolution set to a value of 60,000. For the analysis of tryptic peptides, survey scans were recorded in the FT mode, followed by data-dependent collision-induced dissociation (CID) of the 7 most intense ions in the linear ion trap (LTQ). Raw data were searched with MASCOT (Matrix Science, London, UK) against a UniProt mouse database. The search parameters included variable modifications of 57.02146 Da (carboxyamidomethylation) for Cys, 15.99491 Da (oxidation) for Met and 0.984016 Da (deamidation) for Asn and Gln. The search parameters were as follows: a maximum of 2 missed cleavages; initial precursor ion mass tolerance of 10 ppm; and fragment ion mass tolerance 0.6 Da.

### Statistical analysis

Statistical significance was measured using Student's unpaired *t*-test. Survival curves were compared using the log-rank test. In all of the analyses, *p*-values equal to 0.05 served as the limit of significance. Calculations were performed using GraphPad Prism software.

## Results

### *Y. pestis* EV76 provides immediate robust protection against plague

**Figure 1:**
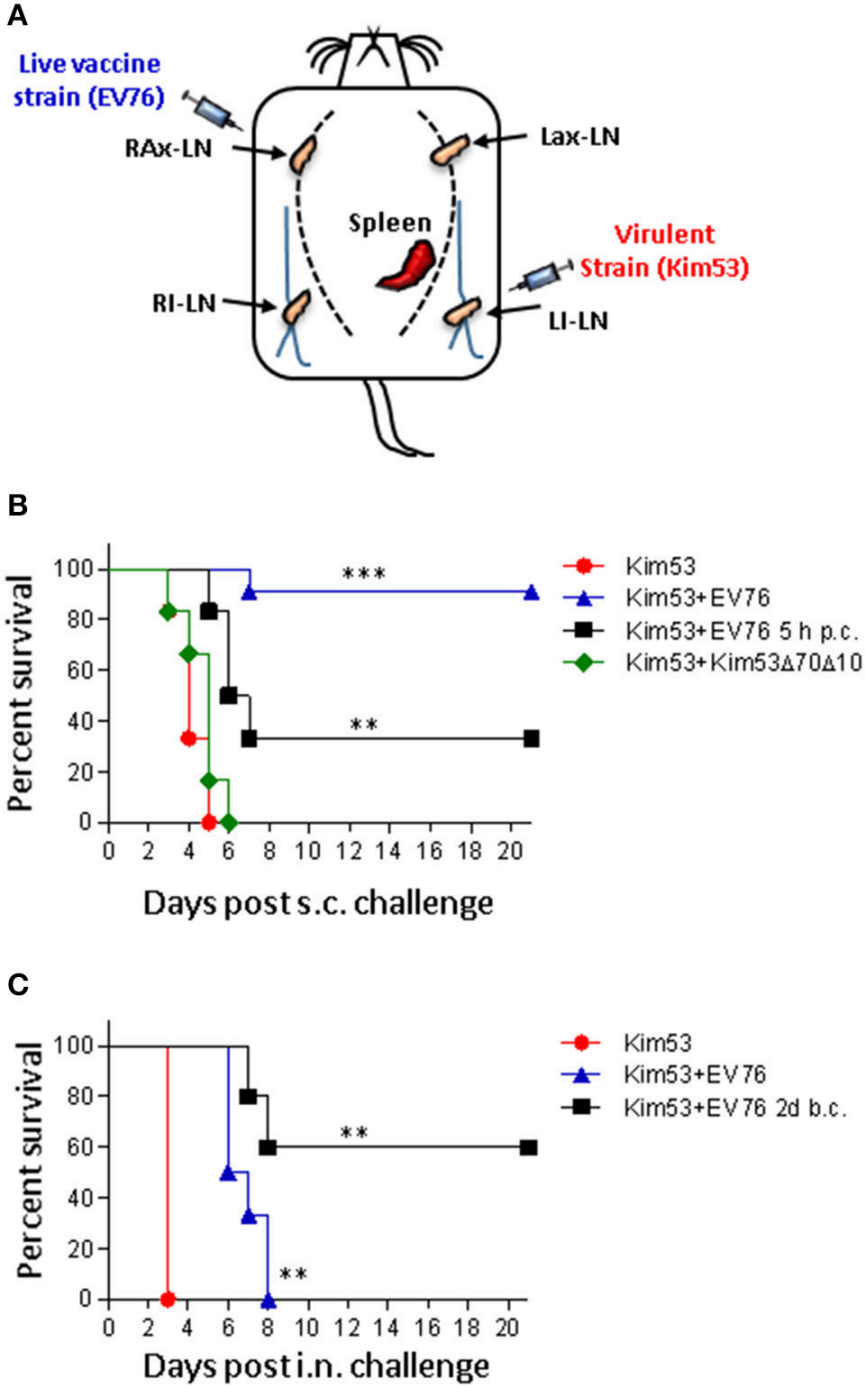
The EV76 strain confers rapid protection against bubonic and pneumonic plague. **(A)** Schematic illustration of the injection sites of the EV76 vaccine strain and the virulent *Y. pestis* Kim53 strain. RAx-LN (right axillary lymph node), LAx-LN (left axillary lymph node), RI-LN (right inguinal lymph node), and LI-LN (left inguinal lymph node). **(B)** Kaplan-Meier survival profiles of mice challenged s.c. in the lower left side with 100 CFU of the virulent *Y. pestis* strain with concomitant immunization in the upper right side with 1 × 10^7^ CFU of the EV76 strain (blue triangles), with 1 × 10^7^ CFU of the Kim53Δ70Δ10 attenuated strain (green diamonds), or with saline (red circles). The black line represents the survival profiles of mice immunized with 1 × 10^7^ CFU of the EV76 strain 5 h after s.c. challenge (p.c.) with the virulent Kim53 *Y. pestis* strain (100 CFU). **(C)** Mice were infected i.n. with 10^4^ CFU of the virulent Kim53 *Y. pestis* strain and were then immediately infected s.c. with the EV76 strain (blue triangle) or with saline (red circle). The black line represents the survival of mice immunized s.c. with 1 × 10^7^ CFU of EV76 2 days before i.n. challenge with 1 × 10^4^ CFU of Kim53 (b.c.); *n* = 6 to *n* = 11 mice per group in at least 2 independent experiments. The *p*-values were calculated using Kaplan-Meier analysis with a log-rank (Mental-Cox) test compared to sham controls. ^**^p < 0.01 and ^***^p < 0.001.

To discover novel innate immune responses that could result in the rapid onset of protective immunity against infection, we evaluated the ability of the live attenuate *Y. pestis* EV76 strain to induce protection against subcutaneous (s.c.) lethal challenge with the fully virulent *Y. pestis* Kimberley53 strain (Kim53), when both strains were administered concomitantly to mice. Accordingly, the mice were infected by s.c. injection of a single dose (1 × 10^7^ CFU) of the attenuated strain and were immediately challenged subcutaneously with a lethal dose of 100 LD_50_ of the virulent strain. To avoid possible direct interaction between the two co-administered strains, the attenuated strain was injected in the upper right back close to the right axillary lymph node (RAx-LN), whereas the virulent strain was injected in the lower left back, close to the left inguinal lymph node (LI-LN). Control mice were injected with saline and were challenged with the virulent *Y. pestis* strain (see scheme in Figure 1A).

As shown in Figure 1B, all of the control mice died within 5 days of the challenge. In contrast, simultaneous co-administration of the attenuated and virulent strains provided a very high protection level of 91%. This result clearly indicated that the EV76 strain was able to elicit an unforeseen, rapid and potent protective response against plague, strongly suggesting that upon immunization, some unknown protective mechanisms manifested prior to the establishment of an adaptive protected state. Simultaneous co-administration of the virulent *Y. pestis* strain with 1 × 10^7^ CFU of another attenuated *Y. pestis* strain (Kim53Δ70Δ10, which lacks the plasmids that carry major *Y. pestis* virulence determinants), could not protect the infected mice (Figure 1B). This observation indicated that the early protective response promoted by EV76 could not be attributed merely to the large amount of bacteria administered but to a particular feature of this strain, and/or to its relative ability to replicate and disseminate in the host.

To examine whether protection could also be achieved by post-challenge administration of the EV76 strain, the mice were injected with EV76 5 h post-challenge. Under this condition, a significantly increased survival rate of 34% was also observed (Figure 1B).

In light of these results, we addressed the question of whether EV76-administration might promote rapid protection against pneumonic plague, which is considered to be a more challenging and severe manifestation of the disease. Accordingly, the mice were challenged intra-nasally (i.n.) with a lethal dose of the virulent *Y. pestis* strain 2 days after s.c. administration of the EV76 strain. As seen in Figure 1C, all of the control mice died at 3 days post-challenge, whereas 60% of the immunized mice survived the challenge. When mice were concomitantly infected with both strains, the mean time to death was extended from 3 to 6.8 days (Figure 1C). These results indicated that exposure to the *Y. pestis* EV76 live vaccine strain induced a very potent protective mechanism within a time window of less than 4 days, which is much earlier than the time usually required for the manifestation of an adaptive protective response.

### Co-administration of the protective EV76 strain restricts the growth and dissemination of the virulent *Y. pestis* strain

**Figure 2:**
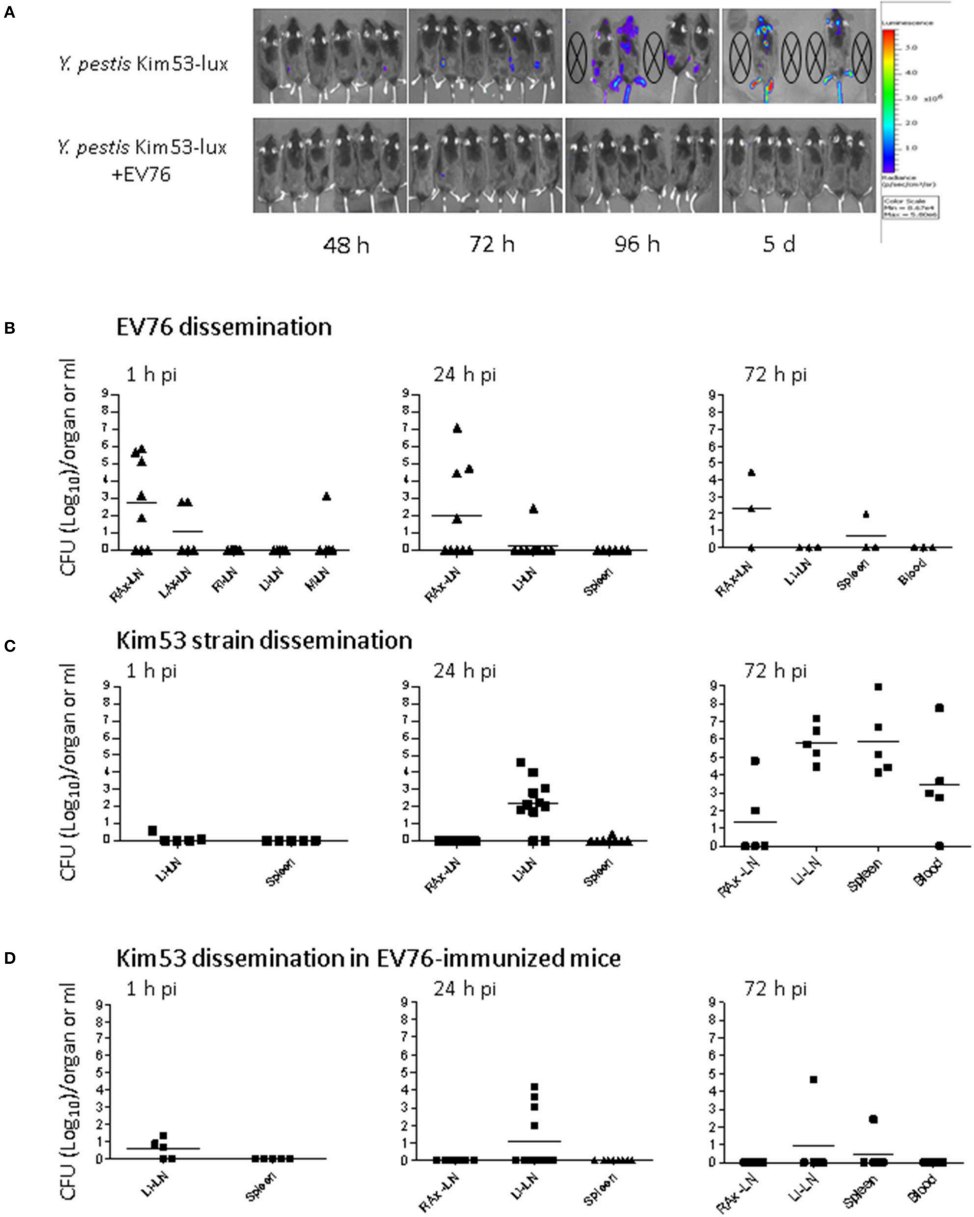
Co-administration of the protective EV76 strain restricts the growth and dissemination of the virulent *Y. pestis* Kim53 strain. **(A)** Dissemination of bacteria in mice was analyzed using an IVIS lumina imaging system at different time points (h, hours; d, days) after s.c. injection of the virulent *Y. pestis* strain Kim53-lux (100 CFU) together with saline (upper panel) or after s.c. injection of *Y. pestis* Kim53-lux (100 CFU) together with 1 × 10^7^ CFU of the EV76 strain (EV76-immunized, lower panel), as schematically described in Figure 1A. **(B)** Mice were infected s.c. with the protective EV76 strain, the virulent Kim53 strain **(C)** or both **(D)**, as described above. At different time points post-infection, the bacterial loads in the draining lymph nodes, spleen and blood were determined; *n* = 3 to *n* = 11 mice per group. Values represent the total bacterial loads in organs (CFU/organ) or the bacterial concentrations in blood (CFU/ml). The limit of detection was 3 CFU. RAx-LN (right axillary lymph node), LAx-LN (left axillary lymph node), RI-LN (right inguinal lymph node), LI-LN (left inguinal lymph node), M-LN (mediastinal lymph node), spleen, and blood. Each dot indicates the value quantified in one animal; bars represent geometric means.

To further characterize the early protective activity induced by the EV76 strain, the effects of the administration of the attenuated strain on the expansion and dissemination of the virulent bacteria in the host were inspected using whole-animal luminescent imaging of individual animals with an *in vivo* imaging system (IVIS). Subcutaneous co-administration of the bacterial strains was performed as described above; however, the virulent strain used for the challenge expressed a bioluminescent tag (Kim53-lux). The animals were monitored daily for bioluminescence emission from 1 day post-infection until death. As depicted in Figure 2A (upper panel), in the non-immunized group, a luminescent signal marking the presence of at least 5 × 10^6^ bacteria (the detection limit) was visible 48 h after the challenge and was limited to the site of injection or to the inguinal lymph node proximal to the injection site. Dissemination of the virulent bacteria into internal organs was first observed 72 h post-challenge. Five days post-challenge, 4 of 6 mice died, and the two remaining mice exhibited strong systemically dispersed luminescent signals. In contrast, mice injected with the protective EV76 strain simultaneously with the virulent strain did not exhibit any luminescent signals throughout the experiment, apart from a single mouse in which a minor and transient signal was observed at 72 h post-challenge at the draining lymph node (Figure 2A, lower panel). No mortality was observed in the immunized group. These results suggested that the protective strain rapidly induced a bacterial inhibitory effect following its administration that blocked the replication and dissemination of the virulent strain early in the course of infection.

To determine the effects of EV76 administration during the early stages of infection when the quantity of virulent bacteria in the host is less than the threshold enabling IVIS-mediated detection, the bacterial loads in organs were quantified by direct CFU counts. The mice were injected s.c. with EV76, with the virulent strain or with both, as above. At different time points post-infection, the tissues of infected mice were harvested, and the bacterial loads in the draining lymph nodes, spleen and blood were determined.

In the mice infected s.c. with 1 × 10^7^ CFU of EV76, bacteria reached the draining RAx-LN proximal to the injection site within 1 h. Then, the bacteria persisted in the lymph node for at least 4 days and typically did not disseminate to other internal organs (Figure 2B). In the mice injected with the virulent Kim53 strain (100 CFU), a small amount of bacteria was found in the draining LI-LN, close to the injection site, within 1 h. The bacterial load at the LI-LN grew rapidly to 6 × 10^5^ CFU (geometric mean) after 72 h and was paralleled by dissemination to the spleen and blood (Figure 2C).

When the virulent strain was co-administered with the protective EV76 strain, it disseminated to the draining LI-LN within 1 h (Figure 2D). However, in contrast to the pattern of distribution observed for the virulent strain in the absence of EV76, by 24 h, the number of bacteria detected in the LI-LN was notably lower, and by 72 h post-challenge, in 4 of 5 mice, the virulent bacteria were cleared from the draining LN and were not detected in the spleen or blood (Figure 2D). These results suggested that EV76 has rapid and systemic effects that inhibit the growth and dissemination of the virulent strain from the draining lymph node to internal organs and indicated that direct interaction between the two strains of the bacteria was not involved in this process. Furthermore, the persistence of EV76 bacteria in the lymph node for at least 4 days post-immunization (Figure 2B) could enable continuous induction of this putative protective mechanism.

### Serum derived from mice following exposure to the protective EV76 strain exerts potent protein-based antimicrobial activity against *Y. pestis*

The results of the dissemination experiments strongly suggested that an antibacterial component was produced upon immunization with the protective EV76 strain, and it possibly migrated through the circulation, affecting propagation of the virulent strain.

**Figure 3:**
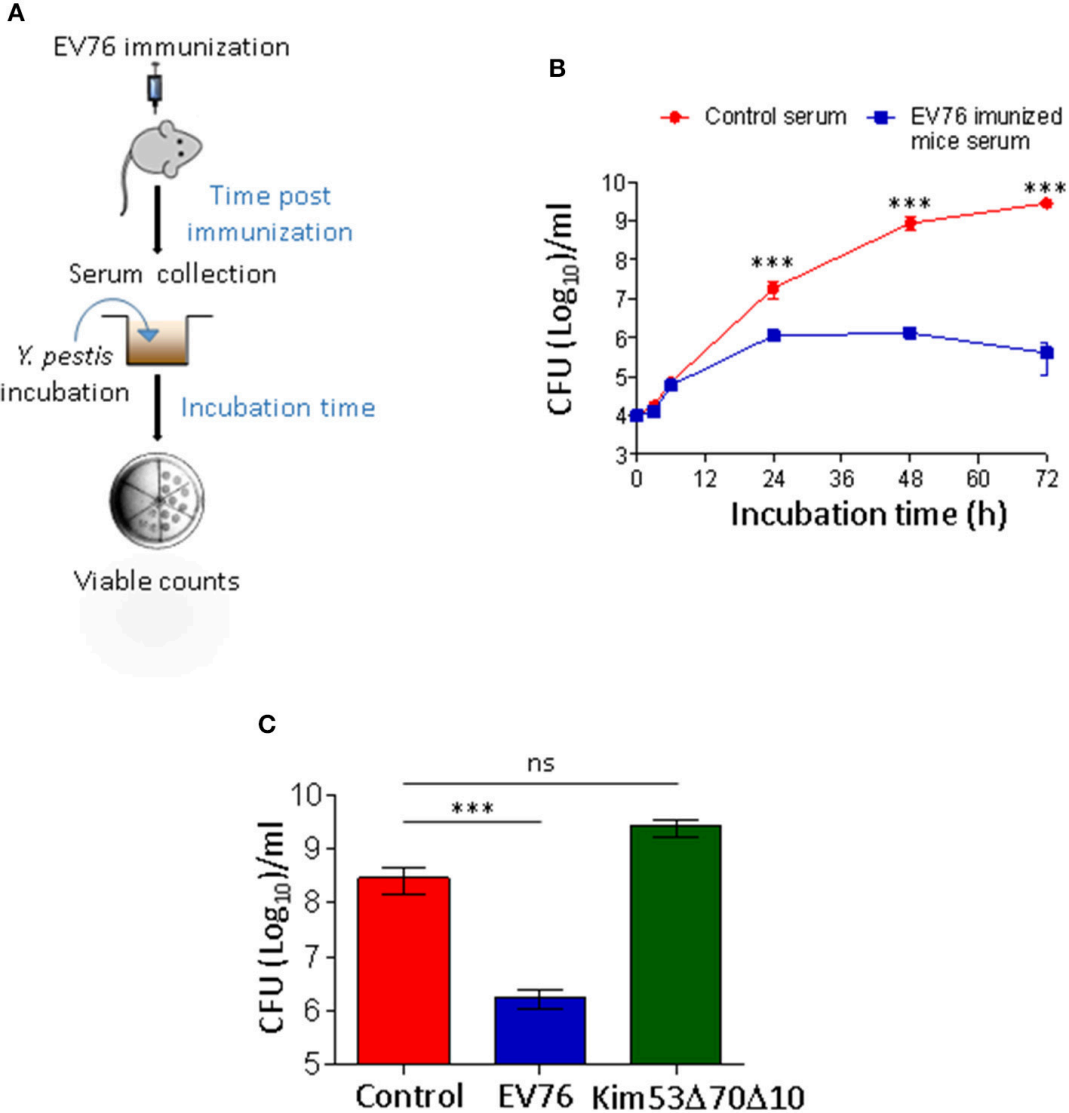
Sera collected from mice immunized with the EV76 strain exhibit growth inhibitory effect against *Y. pestis*. **(A)** Schematic illustration of the *ex vivo* bacterial growth assay; mice were immunized with 1 × 10^7^ CFU of the EV76 strain, and serum was collected at the indicated time points post-immunization (pi). *Y. pestis* bacteria (1 × 10^3^ CFU) were incubated in the presence of murine sera for the indicated incubation times. Growth was monitored by viable counts. **(B)** *Y. pestis* bacteria were subjected to *ex vivo* growth assays as described above, with serum derived from saline-injected mice (red circle) or EV76-immunized mice 24 h p.i. (blue square). Growth was monitored by viable counts following incubation periods of 0–72 h. **(C)** *Y. pestis* bacteria were subjected to *ex vivo* growth assays in the presence of sera derived from control mice or from mice injected with the EV76 strain or the Kim53Δ70Δ10 attenuated strain, as indicated (24 h p.i.). Viable counts were determined after 48 h of incubation. In all of the experiments, the data depict the mean and the standard error of the mean (SEM) of at least 3 individual sera of at least two independent experiments. Statistical significance was measured using Student's unpaired *t*-test with log-transformed values (^***^p < 0.001, and ns, not significant).

To probe a putative non-cellular antibacterial effector, an *ex vivo* growth assay was designed (Figure 3A) in which *Y. pestis* bacteria were subjected to mouse serum from mice 24 h post-immunization (p.i.) with EV76 or to saline-immunized mouse serum (control serum) (Figure 3B). During the first 6 h of incubation, *Y. pestis* bacteria incubated with serum derived from control mice or with EV76-immunized mouse serum exhibited similar growth curves. However, *Y. pestis* growth continued with the control serum for an additional 72 h, reaching a concentration of 2.8 × 10^9^ CFU/ml. In sharp contrast, at 24 h post-incubation, *Y. pestis* growth in the EV76-immunized mouse serum ceased, resulting in significantly lower bacterial concentrations of only 4 × 10^5^ CFU/ml at 72 h. Therefore, the time course of the *Y. pestis* growth inhibition *ex vivo* was correlated with the protection observed *in vivo*. Furthermore, serum collected from mice exposed to the attenuated *Y. pestis* strain Kim53Δ70Δ10, which did not confer protection to mice (Figure 1B), failed to demonstrate inhibition of *Y. pestis* growth *ex vivo* (Figure 3C). Notably, inspection of sera by anti-F1 ELISA clearly demonstrated that the induction of humoral immunity against the immunogenic F1 capsular antigen of *Y. pestis*, which is known to provide anti-plague protection, was observed only on day 8 p.i. with EV76 (anti-F1 IgG titer of 1:1250). Thus, the data suggested that at 24 h p.i. with EV76, the murine serum contained an innate-derived antibacterial component that inhibited bacterial growth.

**Figure 4:**
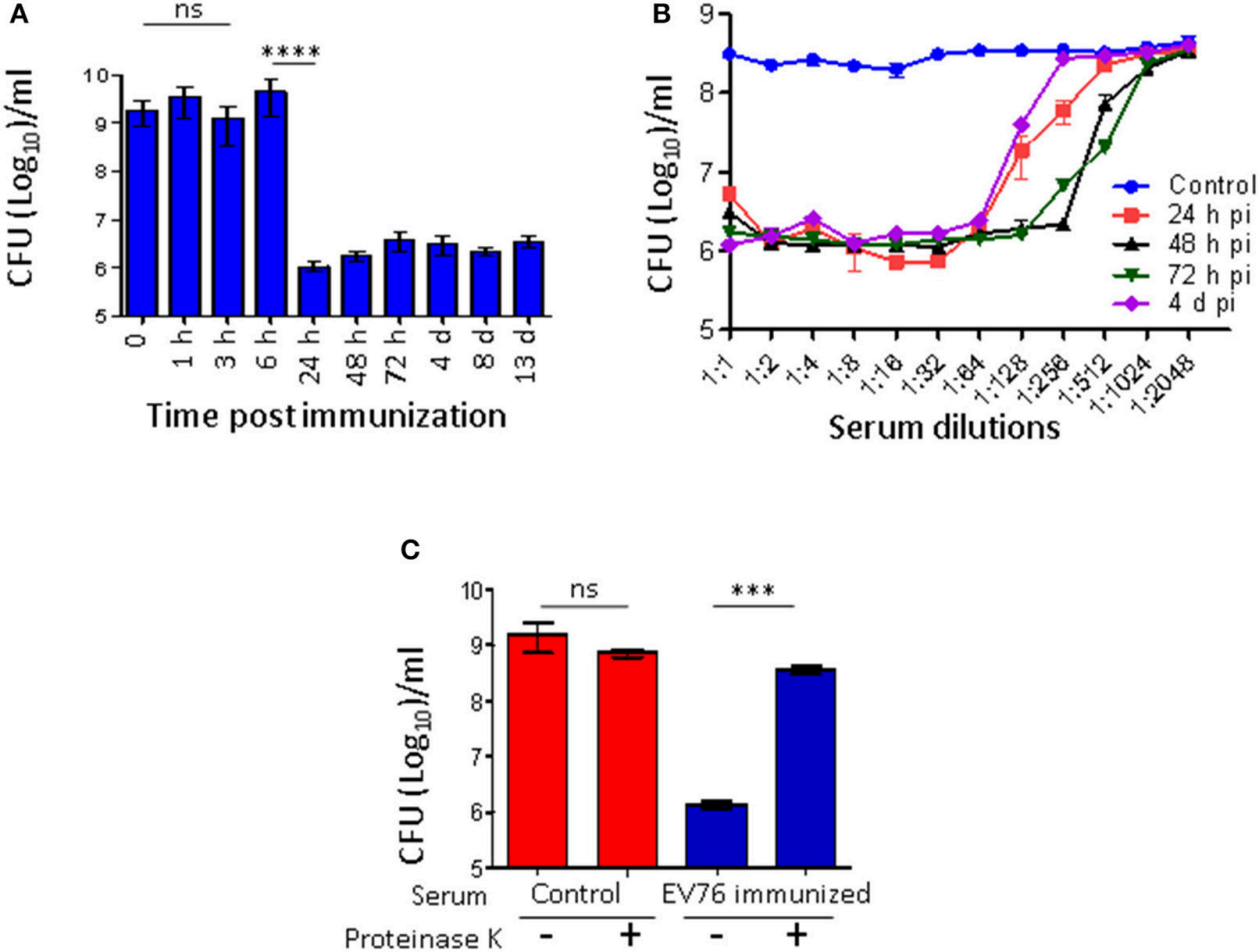
A protenious antibacterial activity is present in the serum 24 h after immunization with the EV76 live vaccine strain. **(A)** Sera collected from EV76-immunized mice at the indicated time points post-infection (1 h–13 d) were analyzed by the *Y. pestis ex vivo* growth assays. **(B)** Serum samples were collected from saline-injected or EV76-immunized mice at the indicated time points p.i. (24 h–4 d), were serial diluted and analyzed for affecting *Y. pestis* growth by *ex vivo* assays. **(C)** Serum derived from control and EV76-immunized mice was subjected to proteinase K digestion and used for *Y. pestis ex vivo* growth assays. In all of the experiments, the data depict the mean and the standard error of the mean (SEM) of at least 3 individual sera, of at least two independent experiments. Statistical significance was measured using Student's unpaired *t*-test with log-transformed values (^****^p < 0.0001, ^***^p < 0.001, and ns, not significant).

To define the kinetics of the antibacterial agent, the mice were immunized with EV76, and serum was collected at different time points post-immunization for *ex vivo Y. pestis* growth assays. Between 1 and 6 h p.i., the serum did not exhibit any anti-growth inhibitory effects (Figure 4A). Inhibition of bacterial growth was observed beginning with serum collected 24 h p.i., and it persisted until day 13. The maximal growth inhibitory effect was observed in serum collected at 48 h and 72 h p.i and the antibacterial activity was found to be highly potent and active even at high dilution (Figure 4B). As depicted in Figure 4C, proteinase K digestion completely eliminated the growth inhibitory effect of the serum, indicating that the antibacterial activity might be attributed to a proteinaceous component.

### Identification of the host proteins hemopexin and transferrin in the serum fraction that inhibits bacterial growth

**Figure 5:**
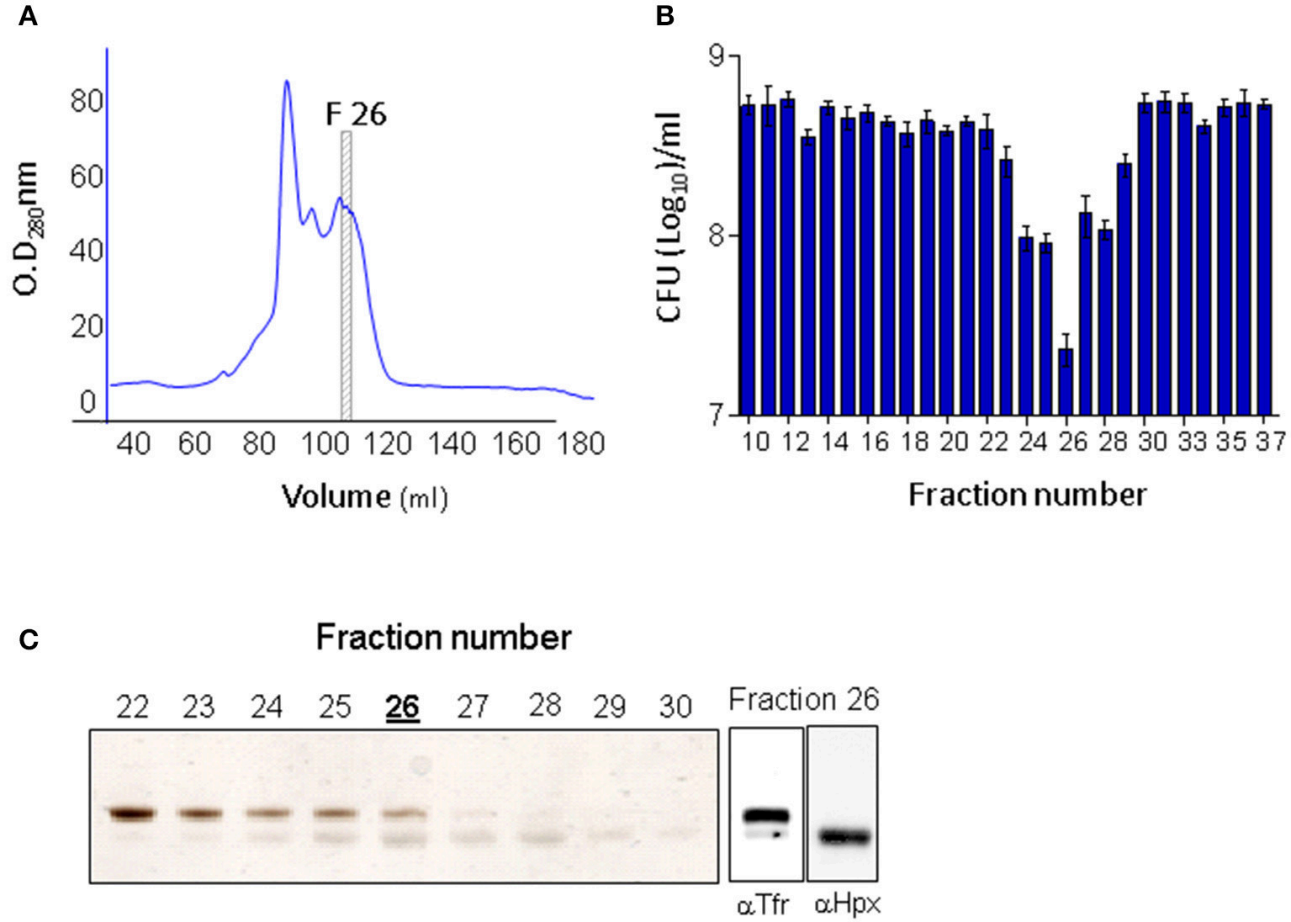
Hemopexin and transferrin are present in the active fraction of EV76-immunized murine serum exhibiting *ex vivo* antibacterial activity. **(A)** Ion-exchange chromatography (second out of two constitutive rounds) of EV76-immunized mice sera. Fraction no. 26 is boxed and marked F26 (it exhibited the highest antibacterial activity, see **B**). **(B)** Antibacterial activity measured in the ion-exchange fractions of the EV76-immunized murine sera. **(C)** Ion-exchange fractions 22–30 inspected by SDS-PAGE visualized by silver staining (left panel). Fraction 26 was subjected to Western blot analysis using anti-transferrin (αTfr) or anti-hemopexin (αHpx antibodies (right).

To identify the antibacterial component in the circulation of EV76-immunized mice, serum samples exhibiting *Y. pestis* inhibitory activity were subjected to HiTrapQ HP anion exchange chromatography fractionations (Figure 5A). The eluted fractions were collected, and their antimicrobial properties were assessed by *ex vivo Y. pestis* growth assays (Figure 5B). Fraction 26 exhibited the maximal growth inhibitory effect. This fraction contained two protein bands visible by silver-stained SDS-PAGE analysis (Figure 5C, left panel). Mass spectrometric tryptic-protein fingerprinting analysis established that the two proteins were host-borne hemopexin (a heme-sequestering protein) and transferrin (an iron-sequestering protein). Using specific antibodies, the identities of these proteins were further confirmed by Western blot analysis (Figure 5C, right panels). Thus, the proteins that seemed to be associated with the inhibitory activity present in the serum of the immunized animals exhibited biological functions involved in iron limitation.

**Figure 6:**
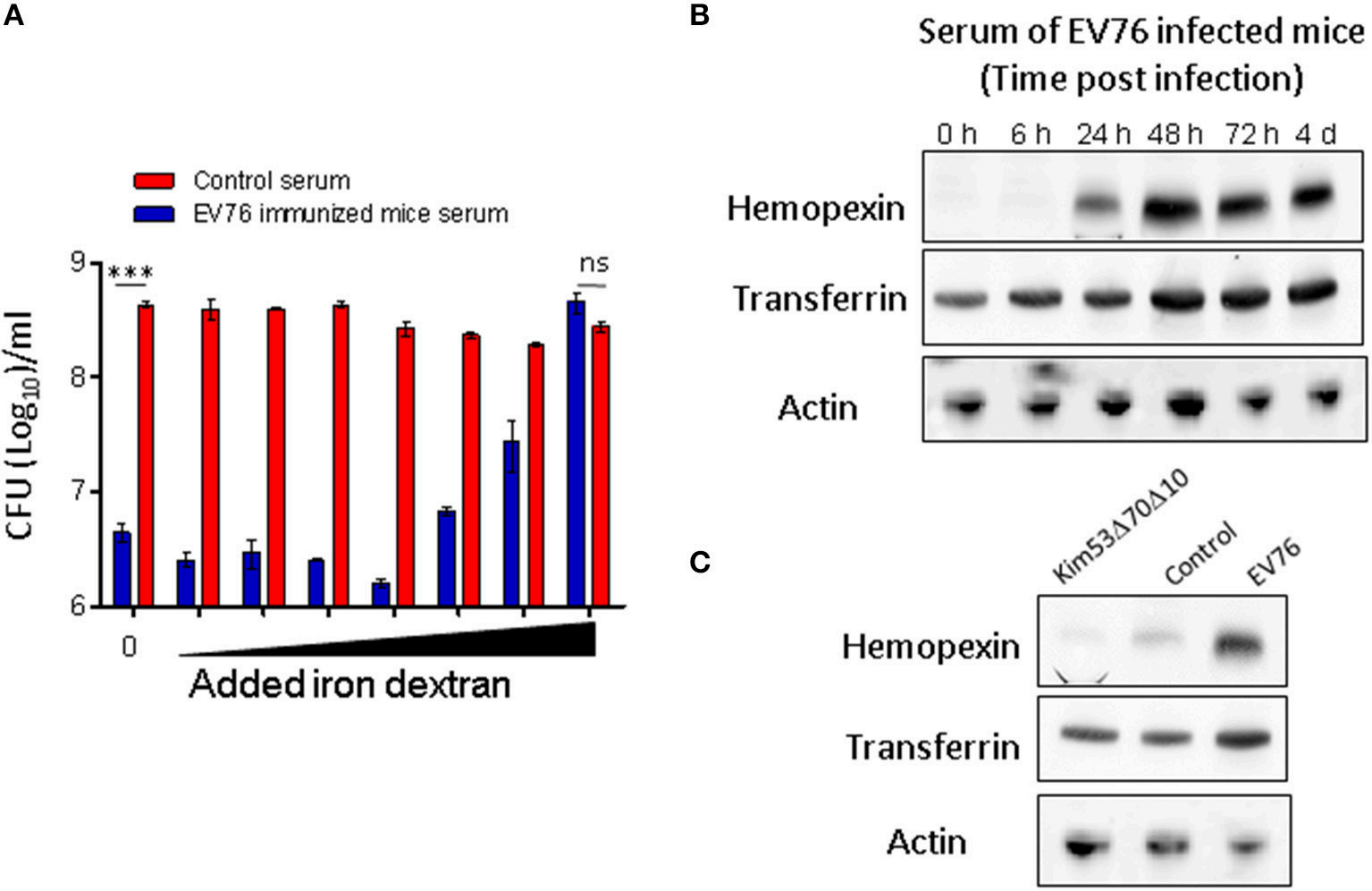
*Y. pestis* growth inhibition by serum collected from EV76-immunized mice is associated with iron deprivation and is induced following EV76 immunization of mice *in vivo*. **(A)** *Y. pestis* incubated in serum (pooled serum samples, *n* = 3) derived from either control mice (red) or EV76–immunized mice (blue) in the presence of increasing amounts of iron (see Materials and Methods). Growth was determined by viable counts. The data depict the mean and SEM. Statistical significance was evaluated using Student's unpaired *t*-test with log-transformed values (^***^p < 0.001 and ns, not significant). **(B)** Pooled sera of mice infected with EV76 at time points from 0 to 4 days post-infection were inspected by Western blot analysis using anti-hemopexin, anti-transferrin or anti-β actin antibodies. **(C)** Pooled sera, collected from mice 24 h post-infection with EV76 or Kim53Δ70Δ10, were examined by Western blot analysis using the indicated antibodies.

To directly investigate the possibility that the antibacterial activity of serum derived from EV76-immunized mice resulted from iron starvation, the *Y. pestis ex vivo* serum-mediated growth inhibition assay was performed in the presence of increasing amounts of iron dextran. Growth inhibition was clearly reversed by iron dextran in a dose-dependent manner (Figure 6A).

### Serum hemopexin and transferrin levels following EV76 immunization

To monitor the expression levels of hemopexin and transferrin following the immunization of mice with EV76, serum samples were collected from EV76-inoculated mice at different time points post-immunization and were subjected to SDS-PAGE and Western blot analysis using specific anti-transferrin and anti-hemopexin antibodies. As shown in Figure 6B, 24 h after EV76 inoculation, hemopexin was dramatically upregulated as compare to control mice. A further increase in hemopexin was detected 48 h following immunization, and this expression level was sustained for at least 4 days. The hemopexin expression pattern strongly correlated with the appearance of antibacterial activity in EV76-immunized mouse serum *ex vivo* (Figures 4A,B). A basal level of transferrin was detected in the serum of non-immunized mice, and its expression level increased slightly upon immunization (Figure 6B). These results suggested that both transferrin and hemopexin were responsible for the antimicrobial activity, however, hemopexin was the major component induced in EV76-immunized mouse serum. The correlation between hemopexin protein levels and the rapid protective response against plague was further substantiated by the observation that injection of the Kim53Δ70Δ10 strain (which did not protect mice from simultaneous challenge with the virulent strain; Figure 1B) did not induce the expression of hemopexin or transferrin in mouse serum (Figure 6C).

## Discussion

The live attenuated *Y. pestis* vaccine strain EV76 confers robust protection against plague due to its elicitation of strong acquired immune responses targeting *Y. pestis* key virulent factors and structural bacterial constituents, such as the F1 antigen (Walker et al., 1953; Tidhar et al., 2009). The establishment of an adaptive immune response is a relatively slow process, requiring several weeks to attain the amplitude needed for manifestation of its protective value. Since post-exposure prophylactic measures are highly relevant in the case of pathogens against which mass vaccination is not routinely conducted or in the case of bacterial pathogens potentially associated with intentional bioterror use, we inspected the effects of EV76 vaccination administered concomitantly with exposure of the experimental animals to a lethal dose of a fully virulent *Y. pestis* strain. Here, we provided evidence that upon s.c. immunization with a single dose of the EV76 live vaccine strain of *Y. pestis*, the mice acquired a protected state almost instantaneously that conferred high resistance to s.c. infection by a lethal *Y. pestis* strain. This effect was prevalent even when the live vaccine was administered several hours post-infection and was also effective when the mice were infected via pulmonary exposure only 2 days after immunization. Early induction of the protected state could not be attributed to an acquired response, which typically requires a much longer time to manifest (in the current study, a minimal 8-day period was measured for the detection of anti-F1 antibodies). To the best of our knowledge, this was the first time that such an immediate and effective stimulation of a protective response to plague by a live vaccine strain was shown. Furthermore, the effect appeared to be due to a particular ability of the EV76 strain because it could not be replicated by the administration of a commensurate dose of another attenuated *Y. pestis* strain. One may speculate that the specificity of the response could be related to the residual ability of EV76 to replicate and disseminate in the host.

Bacterial dissemination data obtained by IVIS-mediated visualization or by direct quantification of the bacterial load in the organs confirmed that the early innate protective response induced by the EV76 strain resulted in complete blockage of the spread of the virulent strain as well as profound limitation of its proliferation. *Ex vivo* assays showed that mouse sera collected 24 h after EV76 immunization (but not from control mice) exhibited a strong anti-*Y. pestis* growth inhibitory effect that was maximal at 48 h post-immunization and was maintained at least until the development of specific antibodies. To address the mechanistic basis for this early systemic antimicrobial activity induced by the EV76 strain, a protein anion exchange chromatographic fractionation of the sera collected from the immunized mice was implemented, enabling mass spectrometric identification of the two host proteins, hemopexin and transferrin, that co-eluted in the antibacterial active fractions. While both proteins appeared to be associated with the antibacterial activity, transferrin was present in control mice sera, and its level was only moderately increased following immunization with EV76. Hemopexin, in contrast, was barely detected in control mice sera, and its expression level was upregulated dramatically soon after EV76 immunization in strong correlation with the antimicrobial activity observed in the *ex vivo* assays with post-immunization sera.

The data therefore strongly suggested that the protective state induced by immunization could be attributed to the biological activities of the host iron and heme-binding proteins. Maintenance of low levels of free iron is a major host innate defense strategy that limits the growth of infectious bacteria and thus establishes nutritional immunity (Weinberg, 1975; Cassat and Skaar, 2013; Parrow et al., 2013; Elphinstone et al., 2016). Accordingly, pathogens have evolved mechanisms to overcome this limitation to gain access to the host iron supply by expressing siderophores and hemophores, which are able to exploit iron that is trapped by host proteins, such as transferrin and hemopexin (Cornelis, 2010; Cassat and Skaar, 2013; Parrow et al., 2013). In the case of *Y. pestis*, extensive studies have documented that deletion of the pigmentation (*pgm*) locus, which is composed of genes encoding for the siderophore-mediated iron acquisition system and hemin storage, is associated with significant virulence attenuation (Perry and Fetherston, 1997; Carniel, 2001). Notably, the EV76 strain itself owes its significantly attenuated phenotype to the absence of the *pgm* pathogenicity locus.

Transferrin was previously shown to participate in host nutritional immunity, which can prevent the availability of free iron in the organism (Sridhar et al., 2000). Induction of hemopexin in response to systemic infection was thought to protect infected host from heme-induced cell damage (Larsen et al., 2010; Medzhitov et al., 2012). Recently, hemopexin was also shown to limit the availability of heme in the context of an IL-22-induced response to systemic infection by enteropathogens (Sakamoto et al., 2017). Our observation that *Y. pestis* growth inhibition appeared to depend on the presence of transferrin and hemopexin and that these effects could be reversed by elevating the amounts of available iron is in good agreement with these studies. Moreover, while in previous studies, iron limitation was associated with the host response to bacterial challenge; our data indicated that this phenomenon, which may not be effectively activated in the case of plague infection, was exploited for the early manifestation of protection by a live attenuated vaccine.

The sequestration of iron promoted by hemopexin and transferrin could explain the early antibacterial effect induced by immunization with EV76. In fact, exogenously elevating the level of the serum iron-binding protein transferrin was suggested to represent a therapeutic approach to preventing iron acquisition by pathogens, such as *Staphylococcus aureus, Acinetobacter, Candida* and *B. anthracis*, consequently improving the survival of the infected host (Rooijakkers et al., 2010; Lin et al., 2014; Bruhn and Spellberg, 2015).

In this study, we showed for the first time that a *Y. pestis* live vaccine brings about a rapid and significant upregulation of the host hemopexin protein. Rapid elevation of hemopexin levels could, in turn, lead to effective heme withdrawal, which, together with preexisting transferrin, limits the availability of iron that is necessary for the growth and dissemination of the virulent strain. This rapid response was followed by the development of additional innate and adoptive immune responses in later stages of the infection. We speculate that the EV76-mediated rapid induction of hemopexin expression involves a bacterial factor that directly or indirectly interacts with host cells after immunization.

The data strongly suggest that the novel EV76-mediated mechanism described in this study may be relevant in the context of other bacterial infections. Yet, at this stage, it is early to speculate on the ability of other live attenuated vaccines to mount similar responses. Studies addressing these issues are currently being conducted in our laboratory.

This study provides the proof of principle for a new concept by which live attenuated vaccines have the potential to induce rapid, broad-range iron nutritional immunity, which could prove beneficial for post-exposure scenarios by enabling the extension of the time window necessary for the development of an acquired specific immune response or by providing additional countermeasures against antibiotic-resistant pathogens.

## Author contributions

AZ and EM conceived and designed the study. AZ, YV, AT, MA, DG, YL, and EM conducted the experiments. AZ, EM, SR, and TC analyzed the data. AZ, EM, and TC wrote the paper.

### Conflict of interest statement

The authors declare that the research was conducted in the absence of any commercial or financial relationships that could be construed as a potential conflict of interest.
